# Latent Variable Forests for Latent Variable Score Estimation

**DOI:** 10.1177/00131644241237502

**Published:** 2024-04-01

**Authors:** Franz Classe, Christoph Kern

**Affiliations:** 1 Deutsches Jugendinstitut e.V., Munchen, Germany; 2 Department of Statistics at Ludwig-Maximilians-University of Munich, Germany

**Keywords:** differential item functioning, item response theory, machine learning, confirmatory factor analysis, factor scores

## Abstract

We develop a *latent variable forest* (LV Forest) algorithm for the estimation of latent variable scores with one or more latent variables. LV Forest estimates unbiased latent variable scores based on *confirmatory factor analysis* (CFA) models with ordinal and/or numerical response variables. Through parametric model restrictions paired with a nonparametric tree-based machine learning approach, LV Forest estimates latent variable scores using models that are unbiased with respect to relevant subgroups in the population. This way, estimated latent variable scores are interpretable with respect to systematic influences of covariates without being biased by these variables. By building a tree ensemble, LV Forest takes parameter heterogeneity in latent variable modeling into account to capture subgroups with both good model fit and stable parameter estimates. We apply LV Forest to simulated data with heterogeneous model parameters as well as to real large-scale survey data. We show that LV Forest improves the accuracy of score estimation if parameter heterogeneity is present.

## Introduction

The use of psychological questionnaires or tests in research usually involves the assumption of a latent variable measured by the questionnaire items. Latent variable modeling provides a versatile toolkit for measuring such latent traits. There are two main areas where latent variables, and particularly latent variable scores, are used: Scaling individuals on a single construct, and estimating latent variable effects in *factor score regression* (FSR) (see Devlieger et al., 2016, 2019) applications.

The first purpose of psychometric latent variable modeling, individual assessment of psychological traits, is a critical component of the cognitive and behavioral sciences (American Psychological Association [APA], 2014). Individual latent variable scores based on observed responses to items of psychological tests are used for psychopathological diagnoses as well as assessment of abilities and personality in occupations and education. However, a major problem is the validity of psychological tests, especially with respect to social minorities (Reynolds et al., 2021). Generally, validity means that a variable measures what it is supposed to measure. Evidence against test validity usually relies on the hypothesis of construct underrepresentation or construct-irrelevant variance, meaning that a variable measures more or less than it should (APA, 2014, p. 12).

Providing evidence for validity usually includes taking into account deviating response behavior in subgroups. Systematic deviations may indicate that the functioning of the scale item differs with regard to certain construct-irrelevant variables. This phenomenon is referred to as measurement noninvariance (Van De Schoot et al., 2015) or differential item functioning (DIF, Bulut & Suh, 2017), and it is present if item parameters differ between subgroups. An item identified as exhibiting DIF is considered biased if the source of variability is irrelevant to the trait being assessed by the test (i.e., construct-irrelevant). However, because any individual characteristic could be defined as construct irrelevant, controlling for item bias may cause real group differences on these variables to be interpreted as bias (see Davies, 2010).

Latent variable scores can be estimated based on item response theory (IRT) (Hartig & Höhler, 2009; Immekus et al., 2019) or confirmatory factor analysis (CFA) (Li, 2016) models (Bhaktha & Lechner, 2021). Practically, construct underrepresentation can be tested for through model fit tests of CFA or IRT models (APA, 2014). Because parameter heterogeneity leads to parameter instability, the assumption of measurement invariance may be investigated via parameter instability tests (Zeileis & Hornik, 2007). However, such a parameter test usually requires a hypothesis about the covariates that negatively affect the parameter stability of a model. In other words, it requires a priori specification of the subgroups for which DIF is suspected.

In recent years, tree-based machine learning methods have been proposed to algorithmically control for DIF in unidimensional IRT models (Komboz et al., 2018; Strobl et al., 2015) through recursive partitioning (Zeileis et al., 2008). Machine learning methods have also been developed to deal with effect heterogeneity in experimental and observational studies (Athey et al., 2019; Athey & Imbens, 2016; Wager & Athey, 2018). As these methods touch on (distinct) aspects of construct validity, they form the ingredients of our approach that focuses on the estimation of unbiased latent variable scores.

We propose *latent variable forest* (LV Forest) for estimating latent variable scores. LV Forest tackles parameter heterogeneity in latent variable models with ordinal and/or numerical response variables by splitting the original data set to reduce parameter heterogeneity. This way, parameter stability with respect to relevant subgroups is established. LV Forest automatically detects relevant subgroups *within* which parameters do not differ w.r.t. construct-irrelevant variables. LV Forest outputs latent variable score estimates from latent variable models with good model fit estimated separately for each relevant subgroup. However, the estimated latent variable scores may differ *between* these relevant subgroups. This way, latent variable scores may be estimated without true-value group differences being misinterpreted as bias. In psychometric testing, the opportunities and the treatment for examinees as well as the assessment and interpretation of test scores need to be comparable across all individuals and groups in a population. For the stages between assessment and interpretation of test scores this means that construct-irrelevant variables as well as construct underrepresentation have no systematic effect on latent variable scores (Xi, 2010). However, relevant subgroups in which this is the case usually have to be defined a priori. LV Forest overcomes this limitation by automatically creating suggestions for structures of relevant subgroups. Thus, the proposal of this method fills a gap in test methodology. LV Forest is based on the *SEMTree* algorithm to ensure computational efficiency (Arnold et al., 2021; A. M. Brandmaier et al., 2013).

LV Forest comes with a number of favorable properties that allow to take complex heterogeneities in the context of latent variable modeling into account. First, LV Forest uses a data-driven approach for detecting groups that are subject to parameter heterogeneity. The researcher only needs to specify a set of construct-irrelevant partitioning variables for which she suspects differences in model parameters. The partitioning variables are then used to algorithmically search for subgroups with conditionally stable parameters in a decision tree-like fashion. This approach is particularly valuable in situations in which a priori specification of all relevant subgroups based on theoretical assumptions may not be feasible and/or is likely to be insufficient. Second, LV Forest computes multiple decision trees to account for the instability of single trees to small changes in the data to detect relevant subgroups robustly. This approach is inspired by random forests and includes random split selection and bagging to increase tree diversity (Breiman, 2001a). Third, decision trees in LV Forest are heavily pruned. This means that subgroups that are subject to parameter heterogeneity are only selected if the model fits the data and the model parameters are stable with respect to a prespecified vector of covariates.

When applying LV Forest in practice, the algorithm iteratively learns which subgroups in the sample are relevant for estimation and uses these subgroups to repeatedly estimate latent variable scores. Thus, LV Forest can be used for latent variable score estimation especially if the assumed latent variable model does not fit the (full) data and/or includes parameter estimates that are unstable with respect to construct-irrelevant covariates. We show that LV Forest estimates accurate scores in complex settings and outperforms naive and singe tree approaches in simulations.

In section “Combining Factor Analytic Modeling and Item Response Theory,” we describe the methodological background of this paper and how the ideas of IRT and Confirmatory Factor Analysis (CFA) can be merged. In section “Parameter Heterogeneity,” the issues of parameter heterogeneity are described and the M-fluctuation test is introduced. In section “Tree-based Machine Learning,” we briefly introduce tree-based machine learning methods and how the algorithmic modeling perspective can be used to account for heterogeneity. Subsequently, our LV Forest approach is described (section “LV Forest”). In sections “Simulation” and “Real Data Application,” simulations as well as an empirical application of LV Forest with survey data are presented. The advantages and limitations of the proposed method are discussed in section “Discussion.”

## Latent Variable Modeling and Score Estimation

Stochastic models which specify the relationship between individual responses to items with a limited amount of response categories and an underlying continuous latent variable are consolidated under the term IRT. Note that IRT was originally developed to examine the response process of individuals. *Confirmatory factor analysis* (CFA), however, is commonly used to formulate assumptions about items within a model that is supposed to reflect a common unobservable phenomenon. The adequacy of these assumptions is usually tested for by testing model quality (Bean & Bowen, 2021). However, modern estimation methods merge the two traditions of latent variable modeling so that certain variants of CFAs are equivalent to an IRT model (Kamata & Bauer, 2008; ten Holt et al., 2010). This means that IRT models may be used for scale evaluation, that is, to determine whether a set of items measures a latent variable. The advantage of an IRT approach is that it better maps the response process to ordinal or dichotomous response variables.

### Combining Factor Analytic Modeling and Item Response Theory

Usually, in IRT models, a latent variable represents the ability of the respondent. This ability is assumed to underlie the response behavior (Steyer & Eid, 2013). In the following, we refer to this latent variable as η . In the multidimensional GRM (see Immekus et al., 2019; Samejima, 1969), a multidimensional IRT model (MIRT) for graded responses which can cover various model structures, several latent variables are measured by response variables $Y_{i}\;\forall \;i=1,\ldots ,m,$ with ordered response categories. The latent variables are comprised in the vector η . This means that the probability of answering in a category *smaller or equal to* a certain ordered category $k_{i}$ depends on the (multidimensional) distribution of the latent variables. This relationship is described by the *cumulative category response function*, that is the η-conditional probability function:

(1) $$P\left(Y_{i}\;\geq \;k_{i}\;|\;\eta \right)=\Phi ({\beta \prime }_{i}\eta -\alpha_{\mathrm{ik}}).$$

The link function Φ is the distribution function of the standard normal distribution. The *threshold parameter* $\alpha_{\mathrm{ik}}$ may be interpreted as the item-category-specific intercept whereas the *discrimination parameters* $\beta_{\mathrm{ij}}$ , that make up the p×1 vector $\beta_{i}$ , can be interpreted as the slope parameters of the multidimensional probability function in Equation 1.

It is possible to efficiently estimate MIRT parameters via CFA modeling. This means that assumptions of an MIRT model can be translated into a special CFA model and parameters can then be estimated in a computationally efficient manner that is common in the CFA framework (limited information approach, see Li, 2016). For this, a continuous, normally distributed latent response variable $Y_{i}^{*}$ is assumed to underlie each nonnumerical observed response variable/endogenous variable $Y_{i}$ . The relation between the latent response variable $Y_{i}^{*}$ and the (multidimensional) distribution of the latent variables is described by the conditional expectation function:

(2) $$E\left(Y_{i}^{*}\;|\;\eta \right)={\beta \prime }_{i}\;\eta .$$

Note that in this model, the *discrimination parameters* $\beta_{\mathrm{ij}}$ are equivalent to the factor loadings in a CFA model. In the factor analytic approach to MIRT modeling, the latent response variable $Y_{i}^{*}$ of item *i* is related to the observed categorical response variable $Y_{i}$ via a threshold relation, that is

(3) $$Y_{i}=k_{i}\;\mathrm{if}\;\alpha_{\mathrm{ik}}<y_{i}^{*}<\alpha_{i(k+1)}.$$

Using the factor analytic approach makes it possible to estimate MIRT parameters through *weighed least squares* (WLS) estimation (Muthén, 1984). Note that WLS estimation makes it possible to include numerical and ordinal endogenous variables within one model. For a numerical response variable $Y_{i}$ , the basic factor analytic model is

(4) $$Y_{i}=\pi_{i}+{\beta^{\prime }}_{i}\eta +\epsilon_{i},$$

where $\pi_{i}$ is the intercept and $\epsilon_{i}$ is the residual variable for item *i*. The conditional expectation function $E\left(Y_{i}\;|\;\eta \right)$ is estimated such that the threshold relationship shown in equation 3 is omitted.

For simplicity, we refer to CFA models with continuous and/or categorical variables as well as multidimensional GRMs as *latent variable models* in this paper. In IRT, the location of an individual on a construct and specific item characteristics are the only factors that account for a person’s response (Immekus et al., 2019; Reeve & Fayers, 2005). From this point of view, it is usually desirable to determine the level of a person in relation to the construct. When using the limited information approach to parameter estimation of the CFA framework, one has to create scores to represent each individual’s placement on the latent variable. These *latent variable scores* are estimated from fitted models and can be used as dependent or independent variables in regression analyses (DiStefano et al., 2009).

The latent variable score estimates in $\hat{\eta }$ , however, do not represent a unique solution to the latent variable η . For any single factor η in a model, there is an infinite number of sets of scores that are equally consistent with the model’s parameters. A latent variable score estimate may not even have identical rankings on different sets of factor scores for the same latent variable. Due to this problem, that is referred to as *indeterminacy*, one can regard $\hat{\eta }$ only as an indicator of η that contains measurement error (Bollen, 1989, p. 305). Thus, the degree to which latent scores are interpretable highly depends on the degree of indeterminacy.

The indeterminacy of latent variable scores varies widely across different models, applications and methods for latent variable score estimation. It may depend, for example, on the degree of commonality between latent variables and response variables (Grice, 2001). It is suggested by Grice (2001), to examine the correlational relationship between η and $\hat{\eta }$ (referred to as *validity*) as well as the correlational accuracy among the scores of all latent variables within the model to evaluate the degree of indeterminacy of latent variable scores. This could, for example, be done through simulation studies.

### Parameter Heterogeneity

In MIRT models, DIF occurs when an item- or category-specific parameter depends on covariates of the manifest variables (i.e., response variables). Such covariates may take the form of characteristics of the individuals responding to the items. For example, the difficulty of an item may depend on ethnicity, education, or gender. Conditioning on such covariates is equivalent to analyzing separately certain subgroups defined by different values on these covariates. Similarly, in CFA models the structural parameters determining the relation between latent variables and endogenous variables may differ between subgroups. We refer to between-subgroup differences of parameters in both MIRT and CFA models as *parameter heterogeneity*.

Let Z be the vector of covariates $\left(Z_{1},\ldots ,Z_{R}\right)$ that contribute to parameter heterogeneity. Let $R_{1},\ldots ,R_{H},$ be the subgroups for which there is parameter heterogeneity and let the subgroups be defined as subsets of the covariate space over Z and let the model parameters be different across all subgroups. In this case, the association with a subgroup $R_{h}$ corresponds to the event $\left\{Z\;=\;R_{h}\right\}$ . The model parameters in a subgroup $R_{h}$ are homogeneous.

Controlling for parameter heterogeneity for ordinal dependent variables in latent variable models can be formalized by assuming η-conditional probability functions of the category $k_{i}$ on the response variable $Y_{i}$ given membership to the subgroup $R_{h}$ , that is

(5) $$P^{Z\;=\;R_{h}}\left(Y_{i}\;\geq \;k_{i}\;|\;\eta \right)=\Phi ({\beta \prime }_{\mathrm{ih}}\;\eta -\alpha_{\mathrm{ikh}}).$$

Accordingly, for a numeric response variable $Y_{i}$ , the η-conditional expectation is assumed to depend on membership to the subgroup $R_{h}$ , that is

(6) $$E^{Z\;=\;R_{h}}\left(Y_{i}\;|\;\eta \right)=\pi_{\mathrm{ih}}+{\beta \prime }_{\mathrm{ih}}\eta .$$

If the latent variables are properly defined, the latent variable vector η does not depend on the covariate vector Z within the subgroups $R_{h}\;\forall \;h=1,\ldots ,H$ in which the parameters are homogeneous, only the model parameters do. This shows that parameter heterogeneity is present when the conditional probability of responding to an item (or the conditional expectation of an item) is different for two individuals *with the same ability*, only because of their group membership.

In practice, parameter heterogeneity can be very problematic because the number of relevant covariates may be very large. Also, there is an even greater amount of possible values or value ranges of these covariates for which model parameters may differ. In addition, complex interactions within the covariate vector Z are possible so that subgroups may only be detected by considering several covariates jointly. If parameter heterogeneity remains undetected, group differences with respect to the latent variables could be misinterpreted (Komboz et al., 2018), meaning they may be due to bias not due to real latent variable score differences.

Systematic parameter instability with regard to a covariate $Z_{r}$ can be tested with the generalized M-fluctuation test (Zeileis & Hornik, 2007). The test is applicable for latent variable models that were fitted to a data set via maximum likelihood (ML). The null hypothesis of the M-fluctuation test is rejected if the empirical fluctuation during parameter estimation is improbably large. To represent the empirical fluctuation process, the partial derivatives of the individual log-likelihood function $\ln\;L(y_{j},\hat{\theta })$ are used. For *k* parameters in the latent variable model, this is given by the score function:

(7) $$\psi (y_{j},\hat{\theta })=\left(\frac{\partial \;\mathrm{lnL}(y_{j},\hat{\theta })}{\partial {\hat{\theta }}_{1}},\ldots ,\frac{\partial \;\mathrm{lnL}(y_{j},\hat{\theta })}{\partial {\hat{\theta }}_{k}}\right),\;\forall j=1,\ldots ,n.$$

Summing this function across the sample and maximizing the results yields asymptotically equivalent parameter estimates to limited information maximum likelihood estimation in CFA models for metric variables (maximum likelihood estimation, see Lee & Shi, 2021). Thus, in the estimation process, the score function ψ leads to the parameter estimates $\hat{\theta }$ via the condition $\sum_{i=1}^{n}\psi \left(y_{i},\hat{\theta }\right)=0$ . The M-fluctuation test checks for systematic fluctuations of the scores, ordered with regard to a covariate $Z_{r}$ . If parameter heterogeneity is present, the scores will differ for different subgroups that are defined as subsets of $Z_{r}$ . Thus, a test statistic is derived from the scaled cumulative sum of the ordered scores and critical values are obtained from simulation (Wang et al., 2014). Given multiple covariates $Z_{r}\in \;Z$ , the generalized M-fluctuation test should be applied for all covariates using a Bonferroni-corrected α-level .

## Tree-based Machine Learning

In section “Parameter Heterogeneity,” we introduced the problem of parameter heterogeneity in latent variable models. We assume that reducing parameter instability by conditioning on a set of covariates Z will lead to several latent variable models with stable parameters. However, we must assume that the relation between the model parameters in a latent variable model and the covariates Z could be nonlinear and that associations may be complex. Thus, we need a method for which no hypotheses or assumptions about the functional form of parameter heterogeneity need to be prespecified. In other words, we need an exploratory method that is able to resemble the complex nature of parameter heterogeneity in a latent variable model. For this, we draw on tree-based machine learning methodology.

Machine learning models are considered parts of the *algorithmic modeling culture*. As a counterpart to models from the *data modeling culture*, algorithmic models assume that natural mechanisms, which produce data, are unknown. Data models like latent variable models, however, are stochastic models that are supposed to represent how response variables are *truly* associated with latent variables. Most often though, stochastic models are not complex enough to emulate the true nature of the association between latent variables and response variables (Shmueli, 2010).

In contrast, algorithmic models serve the purpose of predicting new or future observations through flexible modeling with minimal assumptions. Algorithmic models need to be flexible enough to approximate the data generating function while also being robust toward changes in the data used to fit the model. This compromise is referred to as the *bias-variance trade-off* (Hastie et al., 2009). Algorithmic models acknowledge the complex and inconceivable ways that nature produces data. They do not need to be fully interpretable, they rather need to provide accurate information (Breiman, 2001b).

Decision trees represent a popular set of nonparametric machine learning methods that are usually used for prediction of an outcome variable. A predictive model (referred to as a *tree*) is built by recursively partitioning the covariate space over Z into a set of nodes (referred to as *leaves*) in which the outcome is considered homogeneous (Kern et al., 2019).

*Score-based structural equation model trees*, as presented by Arnold et al. (2021), combine tree-based machine learning with latent variable modeling. The algorithm searches through all partitioning variables to find subgroups that differ with respect to the model parameters. The aim is to find nodes in which the model parameters are considered homogeneous. For this, the generalized M-fluctuation test with respect to any of the partitioning variables is performed at every node of the tree. If there is significant parameter instability, the node is eventually split at a point on the covariate with the greatest instability into two locally optimal segments. The split point is identified as the location on a partitioning variable at which splitting maximizes the respective score-based test statistic (Arnold et al., 2021, p. 8). As a result, the model only needs to be fitted once at each node of the decision tree. Thus, score-based structural equation model trees are computationally efficient methods for parameter heterogeneity reduction. For simplicity we refer to them as *SEMTrees*.

For the purpose of iteratively reducing parameter heterogeneity, it is important not to overfit a decision tree. At first, a minimum sample size within the terminal nodes (leaves) of the tree must be established so that parameters for latent variable models can be properly estimated for the subsamples in the terminal nodes. Then, only splits that significantly reduce parameter heterogeneity (according to the generalized M-fluctuation test) should be performed, otherwise spurious parameter heterogeneities may be induced for the models in the terminal nodes.

A popular extension to single decision trees is random forests. They are purely predictive methods where the true functional form of the relationship between input and response variables is assumed to be unknown before the procedure is applied and the function approximated by random forest is not directly interpretable. The predictions of a random forest, however, are likely to be more accurate than the predictions of most data models (Fife & D’Onofrio, 2021; Shmueli, 2010). If we acknowledge that nature produces data in complex and inconceivable ways, the approximation through a nonstochastic but accurate function by random forest might be preferable compared with data models.

Random forest methodology can be tailored to serve other purposes. For example, *SEM forests* by A. Brandmaier et al. (2016) can be used for selection of variables that predict differences across individuals w.r.t. parameters in Structural Equation Models (SEMs). The method can also be used for outlier detection and clustering. Another method that extends Breiman’s random forest algorithm is the causal forest approach (see also Athey et al., 2019; Athey & Imbens, 2016; Wager & Athey, 2018) that is used for the estimation of individual treatment effects. Given such tailored extensions, tree-based machine learning methods are being applied more commonly in the social science and survey research context (Buskirk, 2018; Kern et al., 2019).

## LV Forest

**Figure 1.**
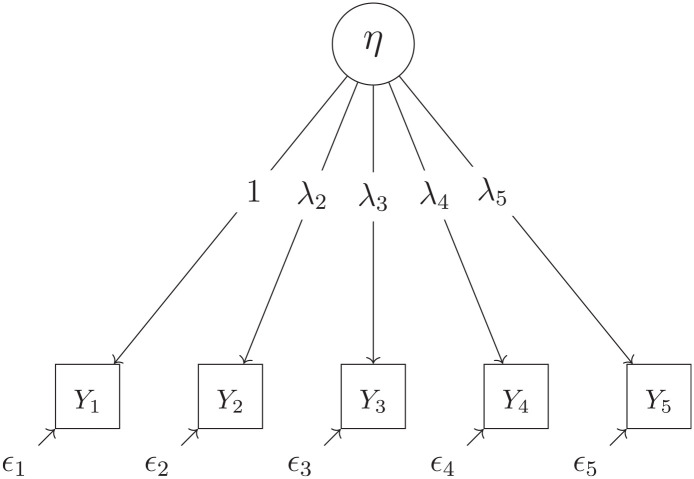
Univariate GRM Model. Item 1 serves as reference item so that $\lambda_{1}$ is fixed to 1.

We develop a tree-based algorithm for latent variable score estimation: LV Forest. The proposed algorithm is outlined in Figure 1. We begin our considerations with the assumption that the parameters of the proposed latent variable model are not equal for all participants in the population. Parameter heterogeneity in the latent variable model may imply unintended influence of construct-irrelevant variables on the relations within the model. Furthermore, we presume that the proposed latent variable model does not fit the data equally well for all subgroups of the population. With the proposed algorithm, we aim to detect subgroups relevant to bias in estimated latent variable scores, and only latent variable models that fulfill conditional independence from construct-irrelevant variables as well as achieve adequate model fit are chosen for latent variable score estimation. This way, we establish both unbiasedness with respect to construct-irrelevant variables in latent variable score estimation and latent variable scores are not estimated with an underrepresented model. We combine the limited-information approach for parameter estimation (section “Combining Factor Analytic Modeling and Item Response Theory”) and the SEMTree algorithm (section “Tree-based Machine Learning”) to efficiently compute an ensemble of decision trees, in which each tree reduces parameter instability. We then prune the resulting trees to detect subgroups in which the model fits the data and the parameter estimations are stable. Note that we do assume that the proposed latent variable model fulfills the criteria for latent variable score determinacy (Grice, 2001, section “Latent Variable Modeling and Score Estimation”).

First, an SEMTree (section “Tree-based Machine Learning”) is grown. Note that for the computation of ψ , which is necessary for the generalized M-fluctuation test (see section “Parameter Heterogeneity”), we need to fit the model with the maximum likelihood (ML) estimator. We then re-assess parameter instability for all construct-irrelevant variables $Z_{1},\ldots ,Z_{R}$ using the M-fluctuation test. Second, the latent variable model is re-fit in each terminal node of the tree. The parameter and model fit estimates in the terminal nodes of the decision tree are calculated using the distribution free weighted least squares (WLS) estimator. Using only the models in the terminal nodes of the tree that fulfill the criteria for model fit and stability of parameter estimates, latent variable scores are then computed via empirical Bayes estimation. For the computation of these Empirical Bayes Modal (EBM) scores, information about response patterns and model parameters are combined with a prior distribution to obtain a posterior distribution. This method is still appropriate if there are categorical or ordinal response variables and it has performed well in simulations (Bhaktha & Lechner, 2021).

We might say that the decision trees in the ensemble are heavily “pruned,” leaving only those leaves that are most likely to contain models that are adequate for latent variable score estimation. Specifically, this means that, we exclude terminal nodes for which (a) the proposed model does not fit the data, and (2) the model’s parameters are instable w.r.t. the covariates. For (a), an RMSEA-cutoff value is defined (Hu & Bentler, 1999; Schermelleh-Engel et al., 2003) and all models that exceed this cutoff are excluded. For (b), the generalized M-fluctuation test for parameter instability (Zeileis & Hornik, 2007) is performed. Classe and Kern (2024) show that the performance of the generalized M-fluctuation test for ordinal data is as good as for metric data and thus can be used for ML-based models.

We learn from the machine learning literature that a single decision tree may be vulnerable to small changes in the training data and the set of partitioning variables (Breiman, 2001a). For the most part, this is a consequence of the hierarchical nature of the decision tree (A. Brandmaier et al., 2016; Kern et al., 2019). In addition, if an SEMTree is grown with ordinal data this can lead to inaccuracies in the partitioning process because the ML estimator is used for the computation of the fitted model scores (section “Parameter Heterogeneity”) at the beginning of the tree growing process. For parameter estimation via maximum likelihood, the dependent variables are assumed to be normally distributed. This assumption rarely holds for ordinal data (Li, 2016). We account for the problem of unstable and potentially inaccurate trees by computing several structurally different decision trees and evaluating the compiled results of this ensemble of trees. We use *random split selection* together with *bootstrap aggregating* (*bagging*) to ensure that the decision trees in the ensemble are structurally different from each other. For random split selection, random selections of partitioning variables are made. The selection of partitioning variables is redrawn at every node in a decision tree. The researcher can specify the number of partitioning variables that are drawn at each node and thus determine the variability between trees. Bagging means that subsamples are randomly drawn from the full data to grow an individual decision tree. This process is repeated to build an ensemble of multiple trees.

After computing all trees in the ensemble, the estimated latent variable scores are accumulated for each individual over all relevant subgroups in the tree ensemble. This means that across all relevant subgroups found by the algorithm that contain individual *i*, the scores are averaged.

**Table:** 

**Algorithm 1:** LV Forest
**Parameters:** minimum sample size in terminal node, RMSEA-cutoff, number of trees in ensemble, number of partitioning variables to sample at each node **1 do** **2** fit model for current sample with ML estimator; **3** randomly sample set of partitioning variables; **4** assess item parameter instability though generalized M-fluctuation test with respect to each selected partitioning variable; **5 if** *parameters are instable AND stopping criteria are not met* then **6** detect covariate $Z_{r^{*}}$ with the strongest instability; **7** select unique value as split point that maximizes the score-based test statistic; **8** split node into two subnodes at split point; **9 for** *each node of current tree* **do** **10** continue partitioning process; **11 end** **12 end** **13 else** **14** stop splitting; **15 end** **16 for** *each terminal node* **do** **17** re-assess parameter instability w.r.t. each covariate $Z_{1},\ldots ,Z_{R}$ (all partitioning variables considered); **18** re-fit model for subgroup in terminal node with the WLS estimator; **19 if** *minimum RMSEA-cutoff exceeds AND parameters are stable* **then** **20** estimate latent variable scores for subgroup in terminal node; **21 end** **22 end** **23 while** *number of iteration < number of trees in ensemble;*

For the application of LV Forest, the R function lvforest was written. In summary, it computes an ensemble of SEMTrees, automatically estimates latent variable scores and tests them for independence of potential construct-irrelevant variables. The R implementation of the proposed method and replication materials for all simulations are provided in the following OSF repository: https://osf.io/gs562/?view_only=c5c715e8e1594445884bb5a1dec27406.

## Simulation

### Setup

We test the performance of LV Forest with simulated data. We carried out three simulations. For Simulation 1, the data are simulated based on a simple univariate latent GRM model, that is

(8) $$\begin{array}{c} P\left(Y_{i}\;\geq \;k_{i}\;|\;\eta \right)=\Phi \left(\lambda_{i}\eta -\kappa_{\mathrm{ik}}\right),\\ \forall \;k=1,\ldots ,6,\;\forall \;i=1,\ldots ,5. \end{array}$$

In this model, the variance and mean of the latent variable are estimated. The discrimination parameter pertaining to item 1 (i.e., $\lambda_{1}$ ) is fixed at 1. Also, the first threshold parameter pertaining to the first item $\kappa_{11}$ is fixed at 1. We simulated five items with seven response categories each, thus there are six threshold parameters $\kappa_{\mathrm{ik}}$ for each response variable. The model is shown in Figure 1.

The data set used in Simulation 1 consist of 10 model-compliant subsamples $\left(R_{h}\;\forall h=1,\ldots ,10\right)$ , each with 500 data points. To simulate model-compliant data, first, true latent variable scores $\overset{\cdot }{\eta }$ were simulated. Furthermore, values of the conditional probabilities $P\left(Y_{i}\;\geq \;k_{i}\;|\;\overset{\cdot }{\eta }\right)$ were computed for all categories of all items. On the basis of these conditional probabilities, values for five ordinal response variables with seven categories each were sampled.

In addition, for each of the simulated subsamples, we created one numerical covariate $\left(\mathrm{nu}m_{h}\right)$ ranging from 1 to 200, one ordinal $\left(\mathrm{or}d_{h}\right)$ and one categorical $\left(\mathrm{ca}t_{h}\right)$ covariate with scores on a 5-point scale. These covariates serve as partitioning variables. For each subsample, the range of values on all partitioning variables were fixed, such that

$$\begin{array}{c} R_{h}:=\left\{\mathrm{nu}m_{h}\leq 50\right\}\cap \left\{\mathrm{ca}t_{h}\in \left\{1,3,5\right\}\right\}\cap \left\{\mathrm{or}d_{h}\geq 4\right\}\;\cap \\ \;\left\{\mathrm{or}d_{\mathrm{is}}\leq 3\;\forall \;i\in R_{s}=\left\{\mathrm{nu}m_{h}\leq 50\right\}\cap \left\{\mathrm{ca}t_{h}\in \left\{1,3,5\right\}\right\}\right\},\;\\ \;\forall \;h,s=1,\ldots ,10,s\neq h. \end{array}$$

This means that the values on $\mathrm{nu}m_{h},\mathrm{ca}t_{h}$ , and $\mathrm{or}d_{h}$ are only fixed for those individuals that belong to subgroup $R_{h}$ except of those individuals *i* belonging to any other subgroup $R_{s}$ and happen to fall within the range of values of $\mathrm{nu}m_{h}\cap \mathrm{ca}t_{h}$ to which $R_{h}$ is fixed. Those individuals are fixed w.r.t. $\mathrm{ca}t_{h}$ . This way, given a complete simulated data set, the model-compliant subsamples are recoverable in the terminal nodes of the decision trees of a tree ensemble. It is, however, not possible to recover all model-compliant subsamples in a single decision tree. In simulating the data this way, we want to mimic the complex data structure produced by natural mechanisms.

All input model parameters that were used to simulate the data differ between all subgroups $R_{h}$ (see Tables 1 and 2). This way, overall parameter instability between the model-compliant partitions of the simulated data set is simulated. The simulation is set up such that the model (see Equation 8) fits the model-compliant subgroups very well (see Table 2).

**Table 1.** Input Threshold Parameters for Simulation.

$R_{1}$	i=1	i=2	i=3	i=4	i=5	$R_{6}$	i=1	i=2	i=3	i=4	i=5
${\overset{\cdot }{\kappa }}_{i1}$	0.00	1.54	0.27	−0.45	−1.24	${\overset{\cdot }{\kappa }}_{i1}$	0.00	1.50	−0.17	−1.56	−0.17
${\overset{\cdot }{\kappa }}_{i2}$	0.31	1.73	0.79	0.13	−0.74	${\overset{\cdot }{\kappa }}_{i2}$	0.75	1.94	0.43	−1.15	0.10
${\overset{\cdot }{\kappa }}_{i3}$	0.90	2.08	0.86	0.42	−0.09	${\overset{\cdot }{\kappa }}_{i3}$	1.34	2.06	0.92	−0.58	0.70
${\overset{\cdot }{\kappa }}_{i4}$	1.20	2.40	1.21	0.84	0.35	${\overset{\cdot }{\kappa }}_{i4}$	2.06	2.34	1.47	0.09	1.66
${\overset{\cdot }{\kappa }}_{i5}$	1.47	2.51	1.31	1.20	1.13	${\overset{\cdot }{\kappa }}_{i5}$	2.33	2.47	1.68	0.73	2.07
${\overset{\cdot }{\kappa }}_{i6}$	1.96	2.76	1.69	1.37	1.55	${\overset{\cdot }{\kappa }}_{i6}$	2.95	2.70	2.17	1.49	2.78
$R_{2}$	i=1	i=2	i=3	i=4	i=5	$R_{7}$	i=1	i=2	i=3	i=4	i=5
${\overset{\cdot }{\kappa }}_{i1}$	0.00	1.06	−0.43	−1.03	−0.14	${\overset{\cdot }{\kappa }}_{i1}$	0.00	0.99	1.98	−1.86	2.66
${\overset{\cdot }{\kappa }}_{i2}$	0.25	1.69	−0.03	−0.40	0.20	${\overset{\cdot }{\kappa }}_{i2}$	0.83	1.96	2.43	−0.68	3.89
${\overset{\cdot }{\kappa }}_{i3}$	0.85	1.98	0.11	0.18	0.40	${\overset{\cdot }{\kappa }}_{i3}$	1.49	2.65	2.52	−0.19	4.22
${\overset{\cdot }{\kappa }}_{i4}$	1.20	2.24	0.37	0.94	0.98	${\overset{\cdot }{\kappa }}_{i4}$	1.95	3.73	2.68	0.23	5.14
${\overset{\cdot }{\kappa }}_{i5}$	1.49	2.51	0.69	1.31	1.29	${\overset{\cdot }{\kappa }}_{i5}$	2.46	4.33	2.94	0.84	5.48
${\overset{\cdot }{\kappa }}_{i6}$	2.00	3.25	0.80	1.80	1.73	${\overset{\cdot }{\kappa }}_{i6}$	2.80	5.34	3.08	1.43	6.04
$R_{3}$	i=1	i=2	i=3	i=4	i=5	$R_{8}$	i=1	i=2	i=3	i=4	i=5
${\overset{\cdot }{\kappa }}_{i1}$	0.00	0.60	0.37	1.94	0.78	${\overset{\cdot }{\kappa }}_{i1}$	0.00	0.20	−1.69	1.54	0.00
${\overset{\cdot }{\kappa }}_{i2}$	0.56	1.22	0.78	2.16	1.27	${\overset{\cdot }{\kappa }}_{i2}$	0.64	1.44	−1.23	1.98	0.71
${\overset{\cdot }{\kappa }}_{i3}$	1.11	1.80	0.96	2.30	1.69	${\overset{\cdot }{\kappa }}_{i3}$	1.03	1.63	−0.54	2.12	1.32
${\overset{\cdot }{\kappa }}_{i4}$	1.74	2.15	1.30	2.54	2.34	${\overset{\cdot }{\kappa }}_{i4}$	1.45	2.33	0.23	2.37	1.84
${\overset{\cdot }{\kappa }}_{i5}$	1.91	2.85	1.53	2.70	3.03	${\overset{\cdot }{\kappa }}_{i5}$	1.87	3.16	0.62	2.47	2.25
${\overset{\cdot }{\kappa }}_{i6}$	2.59	3.43	1.92	2.95	3.63	${\overset{\cdot }{\kappa }}_{i6}$	2.45	4.04	1.48	2.64	2.76
$R_{4}$	i=1	i=2	i=3	i=4	i=5	$R_{9}$	i=1	i=2	i=3	i=4	i=5
${\overset{\cdot }{\kappa }}_{i1}$	0.00	−1.02	−0.43	0.33	0.30	${\overset{\cdot }{\kappa }}_{i1}$	0.00	−0.94	0.99	−0.93	−1.39
${\overset{\cdot }{\kappa }}_{i2}$	0.33	−0.49	−0.22	0.56	0.71	${\overset{\cdot }{\kappa }}_{i2}$	0.52	−0.89	1.76	−0.63	−0.74
${\overset{\cdot }{\kappa }}_{i3}$	0.54	−0.19	0.13	0.84	1.23	${\overset{\cdot }{\kappa }}_{i3}$	0.78	−0.71	1.93	−0.07	−0.30
${\overset{\cdot }{\kappa }}_{i4}$	1.07	0.38	0.76	1.17	1.56	${\overset{\cdot }{\kappa }}_{i4}$	1.16	−0.58	2.60	0.34	0.50
${\overset{\cdot }{\kappa }}_{i5}$	1.41	0.59	1.08	1.34	1.85	${\overset{\cdot }{\kappa }}_{i5}$	1.54	−0.41	3.30	0.94	0.77
${\overset{\cdot }{\kappa }}_{i6}$	1.77	1.19	1.52	1.54	2.17	${\overset{\cdot }{\kappa }}_{i6}$	2.08	−0.17	3.60	1.69	1.63
$R_{5}$	i=1	i=2	i=3	i=4	i=5	$R_{10}$	i=1	i=2	i=3	i=4	i=5
${\overset{\cdot }{\kappa }}_{i1}$	0.00	−1.85	1.45	−0.13	0.44	${\overset{\cdot }{\kappa }}_{i1}$	0.00	1.28	1.52	0.51	−0.91
${\overset{\cdot }{\kappa }}_{i2}$	0.32	−1.24	1.76	0.30	1.00	${\overset{\cdot }{\kappa }}_{i2}$	0.64	1.51	2.01	1.45	0.22
${\overset{\cdot }{\kappa }}_{i3}$	0.71	−0.99	1.96	0.83	1.65	${\overset{\cdot }{\kappa }}_{i3}$	0.96	1.81	2.37	1.77	0.60
${\overset{\cdot }{\kappa }}_{i4}$	1.14	−0.33	2.12	1.27	2.14	${\overset{\cdot }{\kappa }}_{i4}$	1.46	2.00	3.01	2.22	1.47
${\overset{\cdot }{\kappa }}_{i5}$	1.38	0.30	2.22	1.68	2.50	${\overset{\cdot }{\kappa }}_{i5}$	1.84	2.30	3.40	2.54	2.01
${\overset{\cdot }{\kappa }}_{i6}$	1.91	1.00	2.49	2.17	3.21	${\overset{\cdot }{\kappa }}_{i6}$	2.09	2.62	4.17	3.27	2.43

**Table 2.** Model Fit Indicators and Input Discrimination Parameters of Simulated Data Sets.

$\chi^{2}$	*p*-value	RMSEA	$\mathrm{Var}(\overset{\cdot }{\eta })$	$E(\overset{\cdot }{\eta })$	${\overset{\cdot }{\lambda }}_{1}$	${\overset{\cdot }{\lambda }}_{2}$	${\overset{\cdot }{\lambda }}_{3}$	${\overset{\cdot }{\lambda }}_{4}$	${\overset{\cdot }{\lambda }}_{5}$
$R_{1}$	0.116	0.039	0.66	0.99	fixed to 1	0.67	0.57	0.80	1.47
$R_{2}$	0.508	0.000	0.67	0.95		0.80	0.58	1.36	0.89
$R_{3}$	0.641	0.000	1.13	1.21		1.20	0.48	0.37	1.17
$R_{4}$	0.178	0.032	0.68	0.77		1.12	1.12	0.66	0.97
$R_{5}$	0.390	0.009	0.54	0.80		1.51	0.50	1.54	1.33
$R_{6}$	0.715	0.000	1.04	1.76		0.39	0.90	1.00	1.19
$R_{7}$	0.285	0.022	1.15	1.67		1.46	0.39	1.23	1.21
$R_{8}$	0.900	0.000	0.68	1.21		1.57	1.46	0.52	1.36
$R_{9}$	0.122	0.038	0.54	1.03		0.40	1.44	1.32	1.51
$R_{10}$	0.513	0.000	0.70	1.25		0.74	1.30	1.21	1.50

We apply LV Forest to the simulated data set and compute a forest of 10000 decision trees. All covariates $\left(\mathrm{nu}m_{h},\mathrm{ca}t_{h},\mathrm{or}d_{h}\;\forall \;1,\ldots ,10\right)$ are included as partitioning variables. The minimum sample size of the terminal nodes of the trees is set to 200, random split selection is set to 2. We set the model fit cutoff to a RMSEA value of 0.05 to make sure that only the decision rules for well-fitted models are considered when estimating latent variable scores.

Latent variable score estimation accuracy is evaluated by comparing the true simulated latent variable scores $\overset{\cdot }{\eta }$ to latent variable score estimates based on different methods: one fitted model for the entire data set, that is, the *naive* model $\left({\hat{\eta }}_{\mathrm{naive}}\right)$ , a single SEMTree, that is, one fitted model for each terminal node of the single tree $\left({\hat{\eta }}_{\mathrm{SEMTree}}\right)$ , LV Forest $\left({\hat{\eta }}_{\mathrm{LVForest}}\right)$ , and distinct models for the simulated subgroups, that is, 10 separately fitted latent variable models $\left({\hat{\eta }}_{\mathrm{dist}.\mathrm{models}}\right)$ . Note that latent variable score estimation using models fitted on the simulated subgroups individually is not possible in practice as usually the subgroups that are subject to parameter heterogeneity are unknown.

For Simulation 2, we simulated 100 data sets in a simplified form of the procedure described above. We simulated data based on an univariate IRT model with eight items with five categories each (instead of five items with seven categories like in Simulation 1). Furthermore, each of the simulated data sets consist of three model-compliant subsamples for each of which one ordinal $\left(\mathrm{or}d_{h}\right)$ and one numerical $\left(\mathrm{nu}m_{h}\right)$ partitioning variable were created. Each of the simulated subgroups consists of 500 data points so the full data set size is n=1500 (instead of n=5000 in Simulation 1). The range of values on these partitioning variables is fixed, such that

$$\begin{array}{c} R_{h}:=\left\{\mathrm{nu}m_{h}\leq 50\right\}\cap \left\{\mathrm{or}d_{h}\geq 4\right\}\;\cap \left\{\mathrm{or}d_{\mathrm{is}}\leq 3\;\forall \;i\in R_{s}=\left\{\mathrm{nu}m_{h}\leq 50\right\}\right\},\;\\ \;\forall \;h,s=1,\ldots ,3,s\neq h. \end{array}$$

Thus, the model-compliant subsamples are recoverable in the terminal nodes of several decision trees, but not in the terminal nodes of a single decision tree. In Simulation 2, we reduce the number of partitioning variables per simulated data set to six (instead of 30 in Simulation 1).

We apply LV Forest to each of the simulated data sets using the same hyperparameters as in Simulation 1, except that we compute 20 trees per ensemble (instead of 10,000 in Simulation 1). Furthermore, we apply LV Forest to each of the simulated data sets and randomly select 5 out of the 6 relevant partitioning variables to be generally available for the computation of the ensemble. This way, we want to find out how the absence of relevant partitioning variables affects latent variable score estimation with LV Forest. Note that this is not random split selection, but it is a simulation scenario in which not all relevant partitioning variables can be used by the algorithm. We also apply a single SEMTree to each of the simulated data sets, fit a separate model for each of the terminal nodes and estimate latent variable scores using these fitted models.

The accuracy of the latent variable score estimations are evaluated by comparing the true simulated latent variable scores $\overset{\cdot }{\eta }$ to five kinds of latent variable score estimates based on: a naive model $\left({\hat{\eta }}_{\mathrm{naive}}\right)$ , a single decision tree $\left({\hat{\eta }}_{\mathrm{SEMTree}}\right)$ , an LV Forest with absence of one relevant partitioning variable $\left({\hat{\eta }}_{part.LVForest}\right)$ , an LV Forest including all relevant partitioning variables $\left({\hat{\eta }}_{\mathrm{LVForest}}\right)$ , and three distinct models fitted on each of the subgroups $\left({\hat{\eta }}_{\mathrm{dist}.\mathrm{models}}\right)$ . In addition, we evaluate the *nonconvergence* rate of each of the five estimation methods on each of the simulated data sets. The nonconvergence rate describes the relative frequency of individuals in a sample for which latent variable score estimation was not possible, for example, because the model fitting process did not converge. Note that in an LV Forest, “nonconvergence” of latent variable score estimation for individual *i* means that *i* is not part of any relevant subgroup found by the forest and thus scores are not estimated.

For Simulation 3, we simulated one data set in a similar way as in Simulation 1, but now the full data set is simulated using a single set of parameters. We simulated the data to fit a univariate model with five response variables with seven response categories each. We simulate three covariates $\left(\mathrm{nu}m_{1},\mathrm{ca}t_{1},\mathrm{or}d_{1}\right)$ with random values. We apply LV Forest to this data set and compute a forest of 10 decision trees. All covariates are included as partitioning variables. The hyperparameters are set to the same values as in Simulation 1.

### Results

The application of LV Forest with the simulated data resulted in a tree ensemble in which 425 out of 10,000 decision trees included at least one terminal node in which the assumed model fits well and the model parameters are stable w.r.t. the partitioning variables. Overall, there are 439 terminal nodes in which these two conditions apply. These terminal nodes remained for the estimation of latent variable scores for the whole sample. On a 20 core, 170GB RAM server, LV Forest took 5.89 hours (353.5 minutes) of computation time.

**Figure 2.**
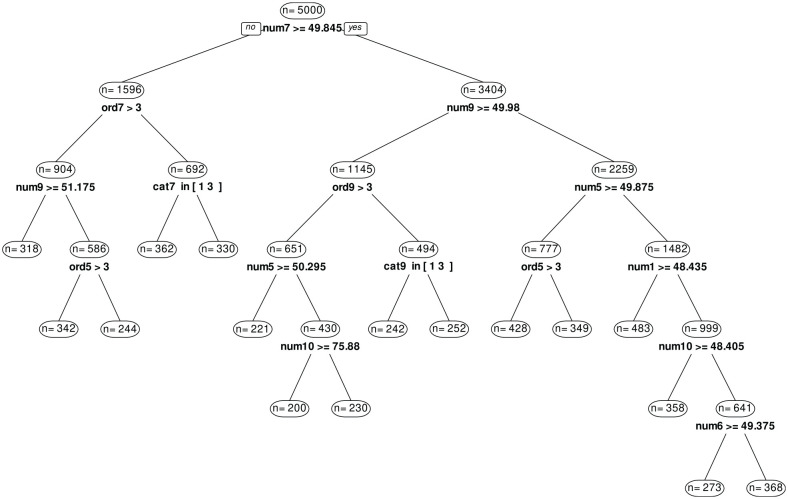
Single SEMTree on Simulated Data (a) Evaluation w.r.t. the correlation of simulated latent variable scores $\overset{\cdot }{\eta }$ with latent variable score estimates. (b) Evaluation w.r.t. nonconvergence of latent variable score estimation for individuals in the sample.

The estimation of the single SEMTree with the simulated data of Simulation 1 took 5.01 minutes on a 20-core, 170GB RAM server. The tree structure is shown in Figure 2. It is obvious that the single SEMTree did not reproduce the simulated subgroup structure. The RMSEA values of the models in the 16 terminal node range from 0.02 to 0.18 but only two of the models have a RMSEA lower than 0.05.

To estimate the naive latent variable scores ${\hat{\eta }}_{\mathrm{naive}}$ , we fit the model in Equation 8 to the whole data set. As suspected, the naive model does not fit the entire data set well (RMSEA=.090,95%C.I.=.080−.101) . The RMSEA values of the distinct subgroups in the data set range from 0.00 to 0.04.

The correlation matrix of the four sets of latent variable score estimates and the true simulated latent variable scores are shown in Table 3. We used Spearman’s rank correlation coefficient because the latent variable score estimations may not follow a normal distribution. The accuracy of latent variable score estimations, that is, the correlations with the simulated latent variable scores $\overset{\cdot }{\eta }$ , are highlighted. The correlation of $\overset{\cdot }{\eta }$ with ${\hat{\eta }}_{\mathrm{naive}}$ (Row 2) is lower than the correlations of ${\hat{\eta }}_{\mathrm{SEMTree}}$ (row 4) and ${\hat{\eta }}_{\mathrm{LVForest}}$ (Row 3) with $\overset{\cdot }{\eta }$ . The correlations of ${\hat{\eta }}_{\mathrm{dist}.\mathrm{models}}$ (row 2) and ${\hat{\eta }}_{\mathrm{LVForest}}$ (Row 3) with $\overset{\cdot }{\eta }$ are very similar and noticeably different from the correlations with ${\hat{\eta }}_{\mathrm{SEMTree}}$ and ${\hat{\eta }}_{\mathrm{naive}}$ with $\overset{\cdot }{\eta }$ . This suggests that latent variable scores estimated by LV Forest may be more accurate than latent variable scores estimated by a single model fitted to the entire data set when there is substantial parameter heterogeneity in the sample. Also, if there is a complex subgroup structure underlying the data, latent variable scores estimated by LV Forest may be more accurate than those estimated by a single SEMTree. Note, however, that the accuracy of latent variable scores depends on the degree of score indeterminacy (see section “Latent Variable Modeling and Score Estimation”). It is still possible that latent variable scores estimated on the basis of a model that fits the data and has stable parameters are inaccurate.

**Table 3.** Correlations of Estimated Latent Variable Scores From Simulation 1.

	$\overset{\cdot }{\eta }$	${\hat{\eta }}_{\mathrm{dist}.\mathrm{models}}$	${\hat{\eta }}_{\mathrm{LVForest}}$	${\hat{\eta }}_{\mathrm{SEMTree}}$	${\hat{\eta }}_{\mathrm{naive}}$
$\overset{\cdot }{\eta }$	1.000				
${\hat{\eta }}_{\mathrm{dist}.\mathrm{models}}$	**0.830**	1.000			
${\hat{\eta }}_{\mathrm{LVForest}}$	**0.816**	0.986	1.000		
${\hat{\eta }}_{\mathrm{SEMTree}}$	**0.761**	0.922	0.950	1.000	
${\hat{\eta }}_{\mathrm{naive}}$	**0.728**	0.899	0.936	0.940	1.000

**Figure 3.**
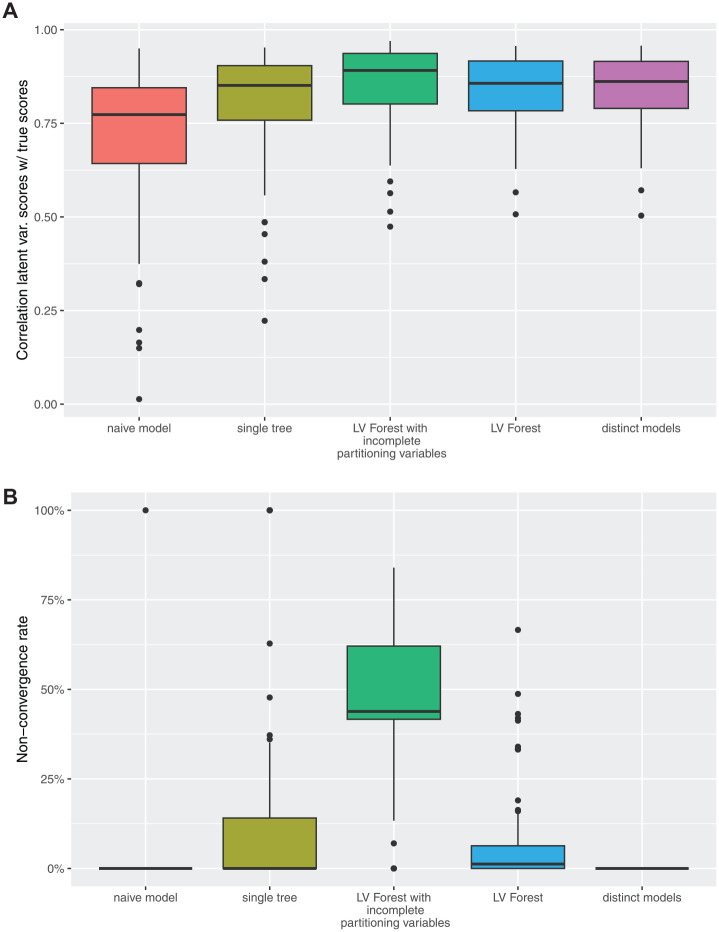
Results of Simulation 2. Latent variable score estimation for 100 data sets based on five different methods. The five methods estimate ${\hat{\eta }}_{\mathrm{naive}}$ (naive model), ${\hat{\eta }}_{\mathrm{SEMTree}}$ (SEMTree), ${\hat{\eta }}_{\mathrm{LVForest}}$ (LV Forest), ${\hat{\eta }}_{\mathrm{part}.\mathrm{LVForest}}$ (LV Forest with incomplete partitioning variables), and ${\hat{\eta }}_{\mathrm{dist}.\mathrm{models}}$ (distinct models).

The results of Simulation 2 in terms of accuracy are shown in Figure 3a and the results in terms of nonconvergence are shown in Figure 3b. The application of the different latent variable score estimation methods on 100 simulated data sets shows that the accuracy of latent variable score estimation based on a naive model $\left({\hat{\eta }}_{\mathrm{naive}}\right)$ is, on average, lower than the accuracy of the other methods. In terms of nonconvergence, the naive model did not estimate latent variable scores on one data set. The SEMTree algorithm did not converge on 4 data sets such that no latent variable score estimations were made. For 27 data sets, the nonconvergence rate is larger than 10% as individual models in the terminal nodes did not converge. For 6 data sets, the accuracy of the latent variable scores ${\hat{\eta }}_{\mathrm{SEMTree}}$ is lower than 0.5. The accuracy of ${\hat{\eta }}_{\mathrm{LVForest}}$ , and ${\hat{\eta }}_{\mathrm{dist}.\mathrm{models}}$ is, on average, higher than the accuracy of ${\hat{\eta }}_{\mathrm{SEMTree}}$ . Also, there are no outliers with accuracy lower than 0.5 for ${\hat{\eta }}_{\mathrm{LVForest}}$ . Overall, ${\hat{\eta }}_{\mathrm{LVForest}}$ seems to be very similar to ${\hat{\eta }}_{\mathrm{dist}.\mathrm{models}}$ in terms of accuracy. However, the analysis of the nonconvergence rates show that there are 17 data sets for which the nonconvergence rate of LV Forest is larger than 10%. Note that if all relevant partitioning variables are included, nonconvergence can be reduced to 0% if more than 20 trees are computed in an ensemble. The analysis of ${\hat{\eta }}_{\mathrm{part}.\mathrm{LVForest}}$ indicates that the high accuracy of LV Forest is not affected if not all partitioning variables are available. However, 95 of 100 data sets exhibit a nonconvergence rate of more than 10% and 30 data sets exhibit a nonconvergence rate of over 50%. This indicates that when using LV Forest, the lack of relevant partitioning variables does not affect the accuracy of the estimated scores, but it does affect the convergence rate and thus the coverage of the scores that are estimated.

Over all 100 samples, the mean computation time of a single SEMTree was 15.17 seconds. The mean computation time of LV Forest was 37.86 seconds. Note that the computations were executed on a 20 core, 170GB RAM server and the trees were computed in parallel.

The results of Simulation 3 show that no splits were performed in any of the 10 LV Forest trees. Thus, in the absence of parameter heterogeneity, the scores estimated by LV Forest are equal to the scores of the naive model.

## Real Data Application

We demonstrate the application of LV Forest using data obtained from the LISS (Longitudinal Internet studies for the Social Sciences) panel administered by Centerdata (Tilburg University, The Netherlands). LISS is a comprehensive longitudinal survey conducted annually, encompassing a wide range of topics such as employment, education, income, housing and personality traits (Scherpenzeel, 2018). For this application, we analyze the data from the first survey wave in 2008. In this wave, 8,722 household members were contacted and 6808 individuals responded. We focus on five items from the satisfaction with life scale (Diener et al., 1985) measuring life satisfaction. We excluded all cases that did not respond on all of the five items which leads to a final sample of n=6626 . The items were rated on a 7-point Likert-type scale. The wording of the items is shown in Table 4.

**Table 4.** Life Satisfaction Scale Items as Asked in the LISS Panel.

**Text:** Below are five statements with which you may agree or disagree. Using the 1–7 scale below, indicate your agreement with each item by placing the appropriate number on the line preceding that item. Please be open and honest in your responding.	
**Item**	**Wording**
i=1	In most ways my life is close to my ideal
i=2	The conditions of my life are excellent
i=3	I am satisfied with my life
i=4	So far I have gotten the important things I want in life
i=5	If I could live my life over, I would change almost nothing

We analyze the data using the same univariate GRM model structure that Simulation 1 is based on (see Equation 8 and Figure 1). First we fit such a model to the whole data set and refer to it as the naive model. We then we apply LV Forest.

For the application of LV Forest, we choose 11 background variables representing the construct-irrelevant variables for our latent variable model. These variables describe the general characteristics of households and household members that participate in the LISS panel. They encode characteristics on the individual level (such as gender, age or civil status) as well as on the household level (such as household income, domestic situation or type of dwelling). The variables are shown in Table 5. We apply LV Forest to the data set using these background variables as partitioning variables and compute an ensemble of 1,000 trees. To reduce computation time and to ensure that LV Forest outputs a manageable number of relevant subgroups w.r.t. post hoc analysis, we set the cutoff RMSEA value to .03. Minimum terminal node size is set to 200 and random split selection to 2.

**Table 5.** Partitioning Variables Used in LV Forest Application With LISS Panel Data.

Variable	Variable namesin LISS data	Level	Type	Values	Value labels
Gender	geslacht	individual	categorical	1	Male
				2	Female
				3	Other
Householdhead livestogetherwith partner	partner	household	categorical	0	No
				1	Yes
Civil status	burgstat	individual	categorical	1	Married
				2	Separated
				3	Divorced
				4	Widow or widower
				5	Never been married
Type ofdwelling	woning	household	categorical	1	Self-owned dwelling
				2	Rental dwelling
				3	Sub-rented dwelling
				4	Cost-free dwelling
				9	Unknown (missing)
Urban characterof place ofresidence	sted	household	ordered	1	Extremely urban
				2	Very urban
				3	Moderately urban
				4	Slightly urban
				5	Not urban
Domestic situation	woonvorm	household	categorical	1	Single
				2	(Un)married co-habitation,without
				3	child(ren) (Un)marriedco-habitation, with child(ren)
				4	Single, with child(ren)
				5	Other
Number ofhousehold members	aantalhh	household	ordered	1	One person
				2	Two persons
				3	Three persons
				4	Four persons
				5	Five persons
				6	Six persons
				7	Seven persons
				8	Eight persons
				9	Nine persons or more
Number of living-at-homechildren	aantalki	household	ordered	0	None
				1	One child
				2	Two children
				3	Three children
				4	Four children
				5	Five children
				6	Six children
				7	Seven children
				8	Eight children
				9	Nine children or more
Age category	lftdcat	individual	ordered	1	14 years and younger
				2	15–24 years
				3	25–34 years
				4	35–44 years
				5	45–54 years
				6	55–64 years
				7	65 years and older
Highest levelof education	oplzon	individual	ordered	1	primary school
				2	intermediate secondary education
				3	higher secondary education/ preparatoryuniversity education
				4	intermediate vocational education
				5	higher vocational education
				6	university
				7	other
Net monthlyincome category	nettocat	individual	ordered	0	No income
				1	EUR 500 or less
				2	EUR 501 to EUR 1000
				3	EUR 1001 to EUR 1500
				4	EUR 1501 to EUR 2000
				5	EUR 2001 to EUR 2500
				6	EUR 2501 to EUR 3000
				7	EUR 3001 to EUR 3500
				8	EUR 3501 to EUR 4000
				9	EUR 4001 to EUR 4500
				10	EUR 4501 to EUR 5000
				11	EUR 5001 to EUR 7500
				12	More than EUR 7500
				13	I don’t know
				14	I prefer not to say

As a sensitivity check, we additionally apply LV Forest with the same data but different partitioning variables. We apply an ensemble with the same hyperparameters as described above while using only the first six variables in Table 5 (geslacht to woonvorm) as partitioning variables.

To illustrate the conditional independence of the estimated latent variable scores, we perform post hoc tests for independence between the estimated latent variable scores and the construct-irrelevant variables within the subgroups found by LV Forest. For this, we apply a test based on the d-variable Hilbert Schmidt independence criterion (Pfister et al., 2018). With this kernel-based nonparametric test, we test for stochastic independence (instead of e.g., linear independence).

As the estimated latent variable scores are accumulated for each individual over all relevant subgroups, the resulting latent variable scores are not expected to be independent of construct-irrelevant partitioning variables for the full sample. Within the relevant subgroups, however, the latent variable scores are expected to be independent of construct-irrelevant variables. Thus, any overall effects of background variables on latent variable scores imply real differences between the relevant subgroups. To analyze such effects on the latent variable scores, we apply regression models using the 11 background variables as individual predictors. We do this for three different outcome variables: the LV Forest scores using all partitioning variables, the LV Forest scores using only a subset of partitioning variables and the latent variable scores estimated with the naive model.

We fit the naive model using the WLS estimator (see Section “Combining Factor Analytic Modeling and Item Response Theory”). The model does not fit the data well (RMSEA=.122,C.I.(95%)=.113−.131) .

In the LV Forest ensemble, 15 trees (1.5% of the ensemble) each generated one terminal node that contained a subsample for which the univariate GRM model fits the data and all parameter estimates are stable w.r.t. all 11 background variables. The model fit indices for all subgroups are shown in Table 6. For these relevant subgroups, latent variable scores were estimated, such that score estimates were available for n=2631 individuals (39.7% of the entire sample). On a 20-core, 170GB RAM server, LV Forest took 32 minutes of computation time. For the LV Forest application with only 6 partitioning variables, score estimates were available for n=1310 individuals. The results of independence tests within the subgroups found by LV Forest using the full set of partitioning variables are shown in Table 7. There is only one construct-irrelevant variable (partner) in subgroup $R_{8}$ that is likely to be stochastically dependent on the estimated latent variable scores of $R_{8}$ . This is the case although the parameter estimates of the fitted model used for latent variable score estimations are stable w.r.t. all construct-irrelevant variables. This result may be due to latent variable score indeterminacy. The results of the other tests indicate that parameter stability of well-fitting models w.r.t. construct-irrelevant partitioning variables generally leads to latent variable scores that are independent of construct-irrelevant partitioning variables given the affiliation to a relevant subgroup. We conclude that these relevant subgroups are found by LV Forest.

**Table 6.** Relevant Subgroups Found in LV Forest LISS Data Application.

Subgroup	Tree	Node	$n_{r}$	RMSEA	Decision rule
$R_{1}$	108	24	215	0.000	{aantalki>1}∩{lftdcat>2}∩{sted<2}∩{geslacht=1}
$R_{2}$	119	8	206	0.000	$\begin{array}{c} \{\mathrm{woonvorm}\notin \{1,4\}\}\cap \{\mathrm{lftdcat}>2\}\cap \{\mathrm{aantalhh}<3\}\cap \\ \left\{\mathrm{nettocat}<3\}\cap \{\mathrm{woning}\notin \{2\}\}\cap \{\mathrm{burgstat}\notin \{1\}\right\} \end{array}$
$R_{3}$	161	11	456	0.022	$\begin{array}{c} \{\mathrm{woonvorm}\in \{2,3\}\}\cap \{\mathrm{aantalki}<1\}\cap \{\mathrm{woning}\notin \{2\}\}\cap \\ \left\{\mathrm{lftdcat}<5\}\cap \{\mathrm{lftdcat}<3\right\} \end{array}$
$R_{4}$	209	10	216	0.000	$\begin{array}{c} \{\mathrm{lftdcat}>2\}\cap \{\mathrm{aantalhh}>1\}\cap \{\mathrm{woning}\notin \{-99,2\}\}\cap \\ \left\{\mathrm{aantalhh}<3\}\cap \{\mathrm{geslacht}=1\}\cap \{\mathrm{burgstat}\notin \{1\}\right\} \end{array}$
$R_{5}$	241	16	264	0.000	$\begin{array}{c} \{\mathrm{geslacht}=1\}\cap \{\mathrm{oplzon}<4\}\cap \{\mathrm{partner}\notin \{0\}\}\cap \\ \left\{\mathrm{burgstat}\notin \{1,2,3,4\}\right\} \end{array}$
$R_{6}$	255	11	205	0.000	$\begin{array}{c} \{\mathrm{nettocat}<3\}\cap \{\mathrm{woonvorm}\notin \{1,4\}\}\cap \{\mathrm{oplzon}>2\}\cap \\ \left\{\mathrm{lftdcat}>2\}\cap \{\mathrm{aantalki}<1\}\cap \{\mathrm{burgstat}\notin \{1\}\right\} \end{array}$
$R_{7}$	355	8	288	0.000	$\begin{array}{c} \{\mathrm{nettocat}<3\}\cap \{\mathrm{aantalki}<1\}\cap \{\mathrm{aantalhh}>1\}\cap \\ \left\{\mathrm{woonvorm}\notin \{2\}\}\cap \{\mathrm{burgstat}\in \{1\}\right\} \end{array}$
$R_{8}$	660	5	336	0.000	$\begin{array}{c} \{\mathrm{aantalki}<1\}\cap \{\mathrm{woning}\notin \{1\}\}\cap \{\mathrm{burgstat}\notin \{3,4\}\}\cap \\ \left\{\mathrm{woonvorm}\notin \{2,3\}\right\} \end{array}$
$R_{9}$	677	21	203	0.011	$\begin{array}{c} \{\mathrm{geslacht}=1\}\cap \{\mathrm{woning}\in \{-99,1\}\}\cap \{\mathrm{aantalhh}>1\}\cap \\ \left\{\mathrm{aantalki}<1\}\cap \{\mathrm{lftdcat}<3\right\} \end{array}$
$R_{10}$	713	12	320	0.026	$\begin{array}{c} \{\mathrm{burgstat}\notin \{1\}\}\cap \{\mathrm{lftdcat}>3\}\cap \{\mathrm{burgstat}\notin \{2,4\}\}\cap \\ \left\{\mathrm{partner}\in \{0\}\}\cap \{\mathrm{woning}\notin \{1\}\right\} \end{array}$
$R_{11}$	745	10	265	0.028	$\begin{array}{c} \{\mathrm{aantalhh}>1\}\cap \{\mathrm{woning}\notin \{-99,2\}\}\cap \{\mathrm{nettocat}<3\}\cap \\ \left\{\mathrm{geslacht}\neq 1\}\cap \{\mathrm{oplzon}<2\}\cap \{\mathrm{woonvorm}\notin \{2\}\right\} \end{array}$
$R_{12}$	790	13	214	0.015	$\begin{array}{c} \{\mathrm{woonvorm}\in \{2,3\}\}\cap \{\mathrm{burgstat}\in \{1,2,3,4\}\}\cap \{\mathrm{aantalhh}<3\}\cap \\ \left\{\mathrm{oplzon}<4\}\cap \{\mathrm{oplzon}<2\}\cap \{\mathrm{sted}<3\}\cap \{\mathrm{geslacht}=1\right\} \end{array}$
$R_{13}$	818	19	285	0.000	$\begin{array}{c} \{\mathrm{woning}\in \{1\}\}\cap \{\mathrm{nettocat}<3\}\cap \{\mathrm{geslacht}=1\}\cap \\ \left\{\mathrm{woonvorm}\in \{1,3,4\}\}\cap \{\mathrm{burgstat}\notin \{1,3,4\}\right\} \end{array}$
$R_{14}$	877	31	208	0.000	$\begin{array}{c} \{\mathrm{aantalhh}>1\}\cap \{\mathrm{woning}\in \{2\}\}\cap \\ \left\{\mathrm{burgstat}\in \{1,2,3,4\}\}\cap \{\mathrm{sted}>3\right\} \end{array}$
$R_{15}$	934	8	200	0.000	$\begin{array}{c} \{\mathrm{woning}\notin \{-99,2\}\}\cap \{\mathrm{aantalhh}>1\}\cap \{\mathrm{woonvorm}\notin \{3\}\}\cap \\ \left\{\mathrm{lftdcat}<4\}\cap \{\mathrm{geslacht}=1\right\} \end{array}$

**Table 7.** Results of Kernel-Based Independence Test: Dependence of Latent Variable (Life Satisfaction) on Construct-Irrelevant Variables Within Relevant Subgroups.

	Gender	Partner	Maritalstatus	Housing	Urbanarea	Dom.situation	No. of HHmembers	No. ofchildren	Age	Education	Net income	$n_{r}$
	geslacht	partner	burgstat	woning	sted	woonvorm	aantalhh	aantalki	lftdcat	oplzon	nettocat	
$R_{1}$	–	0.43	0.71	0.42	0.15	0.43	0.23	0.36	0.72	0.80	0.62	215
$R_{2}$	0.41	0.68	0.30	0.81	0.23	0.35	0.31	0.30	0.77	0.18	0.73	206
$R_{3}$	0.77	1.00	0.76	0.47	0.41	0.53	0.59	0.53	0.24	0.84	0.47	456
$R_{4}$	–	0.47	0.70	0.79	0.18	0.50	0.33	0.52	0.83	0.68	0.41	216
$R_{5}$	–	–	–	0.25	0.77	0.74	0.86	0.88	0.71	0.75	0.77	264
$R_{6}$	0.82	0.67	0.14	0.10	0.12	0.35	0.33	0.32	0.79	0.32	0.35	205
$R_{7}$	0.44	0.73	1.00	0.52	0.87	0.66	0.86	0.78	0.02	0.53	0.23	288
$R_{8}$	0.29	0.00*	0.82	0.46	0.36	0.32	0.34	0.39	0.54	0.30	0.86	336
$R_{9}$	–	0.28	0.77	–	0.39	0.26	0.38	0.18	0.25	0.65	0.35	203
$R_{10}$	0.23	1.00	0.79	0.61	0.44	0.86	0.84	0.67	0.21	0.17	0.55	320
$R_{11}$	–	0.57	0.20	–	0.72	0.57	0.36	0.21	0.16	0.57	0.33	265
$R_{12}$	–	–	0.53	0.02	0.40	0.75	0.73	0.75	0.88	0.12	0.49	214
$R_{13}$	–	0.30	–	–	0.55	0.18	0.50	0.27	0.56	0.44	0.15	285
$R_{14}$	0.73	0.64	0.78	–	0.43	0.66	0.62	0.63	0.70	0.32	0.63	208
$R_{15}$	–	0.25	0.50	0.78	0.73	0.24	0.38	0.16	0.52	0.80	0.54	200

We analyzed the effect of the background variables on the different latent variable score estimations (naive model vs. LV Forest vs. LV Forest with subset of partitioning variables). The results are shown in Table 8. The regression coefficients for the scores of the LV Forest with all partitioning variables indicate a linear effect of two variables (partnership status and domestic situation). For these same variables, the regression coefficients for the scores of the reduced LV Forest show a significant effect. Also, the Spearman’s correlation of the scores of the LV Forest with all partitioning variables and the scores of the reduced LV Forest is 0.99. In contrast, the coefficients for the naive scores additionally show significant effects of four other variables (civil status, age, gender, or urban character of dwelling). This indicates that the effect of partnership status and domestic situation on life satisfaction may not be due to bias. The effect of civil status, age, gender or urban character of dwelling, however, may be due to bias w.r.t. the background variables.

**Table 8.** Regression Coefficients of Covariates on Latent Variable Scores in the Real Data Application.

	Naive model	LV Forest w. all part. vars	LV Forest w. subset of part. vars
Geslacht	0.07*	0.05	0.00
Partner	0.42*	0.14*	0.20*
Burgstat	−0.63*	−0.47	−0.15
Woning	0.54	0.33	−0.12
Sted	0.07*	0.03	−0.03
woonvorm	0.41*	0.11*	0.21*
Aantalhh	0.09	−0.40	−0.41
Aantalki	−0.04	−0.46	−0.48
Lftdcat	0.07*	−0.02	0.06
Oplzon	−0.08	−0.13	−0.07
Nettocat	0.19	0.16	0.67
*N*	6626	2631	1310

The LV Forest with a subset of partitioning variables uses the partitioning variables geslacht to woonvorm.

## Discussion

In this study, we proposed LV Forest, an algorithmic approach to latent variable score estimation. We focused on a setting in which a naive latent variable model is subject to parameter heterogeneity. In this case, fitting a latent variable model and estimating latent variable scores on the basis of this model can lead to false conclusions. The proposed latent variable model may, however, not violate measurement invariance within subgroups that can be defined by covariates. Since tree-based methods have successfully been applied to account for DIF (Komboz et al., 2018; Strobl et al., 2015), we utilized the algorithmic machine learning perspective for handling complex subgroup structures in the context of latent variable score estimation. Assuming that the latent variable scores of a proposed model are determinate (section “Latent Variable Modeling and Score Estimation”), we argue that scores should only be estimated if the latent variable in the proposed model is not underrepresented and independent from construct-irrelevant variables. Construct-irrelevant variables may have an effect on latent variable scores estimated using LV Forest. However, this effect may not be due to bias but due to real differences w.r.t. the latent variable scores between relevant subgroups. We build on the growing body of research that utilizes techniques from the field of machine learning to flexibilize stochastic models when they are confronted with complex covariate structures.

In psychological assessment, bias refers to systematically under- or overestimating personality traits or abilities. Especially cultural bias has been a polarizing issue for many years. The controversy lies in the explanations given for the measured systematic differences in traits and abilities between specific subgroups. Are they based on an interaction of genes and environment (i.e., genuinely different ability levels in different groups) or on different cognitive structures requiring different test characteristics, that is, test bias (see Reynolds et al., 2021). According to Bollen (1989), causality, and therefore validity, is only possible if there are no systematic differences in a latent ability or trait with respect to variables outside of the latent variable model. Thus, if systematic differences between groups are not part of the assumed model, they are attributable to test bias. This way, no real differences of the latent variable scores w.r.t. construct-irrelevant variables are interpretable. As virtually all individual characteristics can be such construct-irrelevant variables, this notion is problematic (see, e.g., Davies, 2010). We propose a solution to this problem by proposing a method to estimate latent variables scores whose subgroup differences w.r.t. construct-irrelevant variables are estimable and interpretable.

Latent variable scores estimated using LV Forest are also very useful when it comes to complex SEMs that include measurement paths between latent variables. In these models, *spurious relations* or *suppressor relations* from response variables to latent variables are likely to occur (Bollen, 1989, pp. 51–53). These unmodelled relations distort the other parameters in the model. Therefore, the estimation of effects between two latent variables should rather be performed via FSR (Devlieger et al., 2019) with LV Forest being used for latent variable score estimation.

We applied LV Forest to simulated data to test whether the method is suitable for finding simulated subgroups based on fitting IRT models with stable parameters. The results show that the method works well for an univariate GRM model. We also show that latent variable score accuracy depends, to some degree, on model fit and parameter stability of a latent variable model. Furthermore, we show that latent variable score estimation via a single SEMTree does not perform as good as LV Forest if the subgroup structure behind the sample cannot be recovered by a single tree. Another advantage of LV Forest is that a 0% convergence rate is very unlikely. However, nonconvergence rates are likely to be larger for LV Forest compared with a naive model. However, if there are not many partitioning variables in the data and/or if the data set is not very large, one might prefer using a single SEMTree over LV Forest to estimate latent variable scores.

Furthermore, we applied LV Forest to real data from a large-scale survey. We analyzed five items measuring satisfaction with life and used background variables to recursively partition the sample. As a result, latent variable scores were estimated for 40% of the sample. When the number of partitioning variables was reduced, scores were only estimated for 20% of the sample. This shows that LV Forest may be limited when it comes to exhaustively estimating latent variable scores for the entire sample. In reality, there may always be individuals for which the proposed latent variable model does not apply and relevant partitioning variables are not measured. Our simulations, however, suggest that the accuracy of LV Forest scores is still high, even given considerable nonconvergence. When this is the case, the researcher may increase the RMSEA-cutoff to reduce the nonconvergence rate, but potentially compromise on latent variable score accuracy.

The fact that the estimated latent variable scores were predominantly $R_{h}-\mathrm{conditionally}$ independent from all construct-irrelevant variables in the real data application shows that controlling for DIF w.r.t. construct-irrelevant variables leads to latent variable scores with no systematic effects regarding construct-irrelevant variables *within* relevant subgroups. That is, within these subgroups, all covariance from construct-irrelevant variables is interpreted as bias. *Between* those subgroups, there may be systematic differences regarding construct-irrelevant variables. These differences can be smaller when fewer partitioning variables and a stricter RMSEA-cutoff are used, that is, when fewer relevant subgroups are found. LV Forest estimates latent variable scores that can be interpreted w.r.t. systematic effects of construct-irrelevant variables without inducing bias.

### Comparison to Related Methods

Another tree-based machine learning approach to identify and account for parameter heterogeneity, which is also applicable to different types of latent variable models, is called *Model Based Recursive Partitioning* (MOB) (Zeileis et al., 2008). MOB is designed to grow single trees that avoid overfitting and bias. The MOB algorithm applies the M-fluctuation test (see section “Combining Factor Analytic Modeling and Item Response Theory”) at every node of the tree. Splitting is only performed if parameter heterogeneity is significant with regard to at least one covariate. The covariate with the lowest p-value is selected for splitting. However, splitting is performed in such a way that the sum of the log-likelihood of the two resulting models is maximized. Thus, as many models have to be fit as there are possible split points on a variable chosen for splitting. This is computationally more expensive than score-based SEMTree.

### Limitations

In the LV Forest framework, we focus on latent variable models that may be subject to parameter heterogeneity. Simultaneously, we claim that we only use models with $R_{h}-\mathrm{conditionally}$ unbiased measurement paths for latent variable score estimation. For this, we test for parameter homogeneity using the M-based fluctuation test. However, it is controversial to rely on this test too much if ordinal response variables are used because ML estimation is necessary for the computation of the test. If categorical response variables are used, the assumption of normality of the response variables may be violated. However, ordinal response variables are relevant for many applications and (Classe & Kern, 2024) showed that the results of the M-fluctuation test can be reliable for ordinal response variables.

Practical limitations stem from the fact that it is impossible in many cases to measure all construct-irrelevant variables that may confound the measurement paths of a presumed model. The scores estimated by LV Forest should be interpreted with regard to the fact that there may still be potential construct-irrelevant variables that were not collected in the study. The simulation showed that the absence of relevant partitioning variables may lead to nonconvergence score estimation for individuals in the sample. Thus, if not all relevant partitioning variables are measured, it may not be possible to estimate unbiased scores for every individual in the sample. We additionally note that large samples sizes are needed for LV Forest to be efficient. The sample needs to be large enough that sample sizes in terminal nodes in complex trees are sufficient to estimate model parameters, as well as to accurately perform M-fluctuation tests. The simulation also showed that if the subgroup structure of a sample is complex, many trees and therefore long computation times are needed. In practice, if an assumed model does not fit the data and/or has unstable parameters it may be viable for the researcher to adjust the model assumptions before turning to LV Forest. We also acknowledge that LV Forest does not return an inherently interpretable model function. Like random forests, LV Forest allows to model highly complex structures of subgroups. However, a direct interpretation of the composition of these subgroups would lead to results that are unlikely to be generally applicable. Our proposed method therefore explicitly focuses on the estimation of latent variable scores.

## References

[bibr1-00131644241237502] American Psychological Association. (2014). Standards for psychological and educational testing. American Educational Research Association.

[bibr2-00131644241237502] Arnold M. Voelkle M. C. Brandmaier A. M. (2021). Score-guided structural equation model trees. Frontiers in Psychology, 11, Article 564403. 10.3389/fpsyg.2020.564403 PMC7875879 33584404

[bibr3-00131644241237502] Athey S. Imbens G. (2016). Recursive partitioning for heterogeneous causal effects. Proceedings of the National Academy of Sciences, 113(27), 7353–7360. 10.1073/pnas.1510489113 PMC4941430 27382149

[bibr4-00131644241237502] Athey S. Tibshirani J. Wager S. (2019). Generalized random forests. The Annals of Statistics, 47(2), 1148–1178.

[bibr5-00131644241237502] Bean G. J. Bowen N. K. (2021). Item response theory and confirmatory factor analysis: Complementary approaches for scale development. Journal of Evidence-Based Social Work, 6, 597–618.

[bibr6-00131644241237502] Bhaktha N. Lechner C. M. (2021). To score or not to score? a simulation study on the performance of test scores, plausible values, and SEM, in regression with socio-emotional skill or personality scales as predictors. Frontiers in Psychology, 12, Article 679481. 10.3389/fpsyg.2021.679481 PMC8554300 34721136

[bibr7-00131644241237502] Bollen K. A. (1989). Structural equations with latent variables (Vol. 210). John Wiley.

[bibr8-00131644241237502] Brandmaier A. Prindle J. Mcardle J. Lindenberger U. (2016). Theory-guided exploration with structural equation model forests. Psychological Methods, 21, 566–582. 27918182 10.1037/met0000090

[bibr9-00131644241237502] Brandmaier A. M. von Oertzen T. McArdle J. J. Lindenberger U. (2013). Structural equation model trees. Psychological Methods, 18(1), 71–86. 22984789 10.1037/a0030001 PMC4386908

[bibr10-00131644241237502] Breiman L. (2001a). Random forests. Machine Learning, 45(1), 5–32.

[bibr11-00131644241237502] Breiman L. (2001b). Statistical modeling: The two cultures (with comments and a rejoinder by the author). Statistical Science, 16(3), 199–231.

[bibr12-00131644241237502] Bulut O. Suh Y. (2017). Detecting multidimensional differential item functioning with the multiple indicators multiple causes model, the item response theory likelihood ratio test, and logistic regression. Frontiers in Education, 2, Article 51.

[bibr13-00131644241237502] Buskirk T. D. (2018). Surveying the forests and sampling the trees: An overview of classification and regression trees and random forests with applications in survey research. Survey Practice, 11(1), 1–13.37201036

[bibr14-00131644241237502] Classe F. Kern C. (2024). Detecting differential item functioning in multidimensional graded response models with recursive partitioning. Applied Psychological Measurement. https://doi-org.emedien.ub.uni-muenchen.de/10.1177/01466216241238743 10.1177/01466216241238743 PMC10993862 38585304

[bibr15-00131644241237502] Davies A. (2010). Test fairness: A response. Language Testing, 27(2), 171–176.

[bibr16-00131644241237502] Devlieger I. Mayer A. Rosseel Y. (2016). Hypothesis testing using factor score regression: A comparison of four methods. Educational and Psychological Measurement, 76(5), 741–770. 29795886 10.1177/0013164415607618 PMC5965529

[bibr17-00131644241237502] Devlieger I. Talloen W. Rosseel Y. (2019). New developments in factor score regression: Fit indices and a model comparison test. Educational and Psychological Measurement, 79(6), 1017–1037. 31619838 10.1177/0013164419844552 PMC6777064

[bibr18-00131644241237502] Diener E. Emmons R. A. Larsen R. J. Griffin S. (1985). The satisfaction with life scale. Journal of Personality Assessment, 49(1), 71–75. 16367493 10.1207/s15327752jpa4901_13

[bibr19-00131644241237502] DiStefano C. Zhu M. Mindrila D. (2009). Understanding and using factor scores: Considerations for the applied researcher. Practical Assessment, Research, and Evaluation, 14(1), 20.

[bibr20-00131644241237502] Fife D. D’Onofrio J. (2021). Common, uncommon, and novel applications of random forest in psychological research. Behavior Research Methods, 55, 2447–2466. 10.3758/s13428-022-01901-9 35915361

[bibr21-00131644241237502] Grice J. W. (2001). Computing and evaluating factor scores. Psychological Methods, 6(4), 430–450. 11778682

[bibr22-00131644241237502] Hartig J. Höhler J. (2009). Multidimensional IRT models for the assessment of competencies. Studies in Educational Evaluation, 35(2–3), 57–63.

[bibr23-00131644241237502] Hastie T. Tibshirani R. Friedman J. (2009). The elements of statistical learning: Data mining, inference, and prediction. Springer.

[bibr24-00131644241237502] Hu L.-T. Bentler P. M. (1999). Cutoff criteria for fit indexes in covariance structure analysis: Conventional criteria versus new alternatives. Structural Equation Modeling: A Multidisciplinary Journal, 6(1), 1–55.

[bibr25-00131644241237502] Immekus J. C. Snyder K. E. Ralston P. A. (2019). Multidimensional item response theory for factor structure assessment in educational psychology research. Frontiers in Education, 4, Article 45. 10.3389/feduc.2019.00045

[bibr26-00131644241237502] Kamata A. Bauer D. J. (2008). A note on the relation between factor analytic and item response theory models. Structural Equation Modeling: A Multidisciplinary Journal, 15(1), 136–153.

[bibr27-00131644241237502] Kern C. Klausch T. Kreuter F. (2019). Tree-based machine learning methods for survey research. Survey Research Methods, 13(1), 73–93. 32802211 PMC7425836

[bibr28-00131644241237502] Komboz B. Strobl C. Zeileis A. (2018). Tree-based global model tests for polytomous Rasch models. Educational and Psychological Measurement, 78(1), 128–166. 29795950 10.1177/0013164416664394 PMC5965621

[bibr29-00131644241237502] Lee T. Shi D. (2021). A comparison of full information maximum likelihood and multiple imputation in structural equation modeling with missing data. Psychological Methods, 26, 466–485. 33507765 10.1037/met0000381

[bibr30-00131644241237502] Li C.-H. (2016). Confirmatory factor analysis with ordinal data: Comparing robust maximum likelihood and diagonally weighted least squares. Behavior Research Methods, 48(3), 936–949. 26174714 10.3758/s13428-015-0619-7

[bibr31-00131644241237502] Muthén B. (1984). A general structural equation model with dichotomous, ordered categorical, and continuous latent variable indicators. Psychometrika, 49(1), 115–132.

[bibr32-00131644241237502] Pfister N. Bühlmann P. Schölkopf B. Peters J. (2018). Kernel-based tests for joint independence. Journal of the Royal Statistical Society: Series B (Statistical Methodology), 80(1), 5–31.

[bibr33-00131644241237502] Reeve B. B. Fayers P. (2005). Applying item response theory modeling for evaluating questionnaire item and scale properties. Assessing Quality of Life in Clinical Trials: Methods of Practice, 2, 55–73.

[bibr34-00131644241237502] Reynolds C. R. Altmann R. A. Allen D. N. (2021). The problem of bias in psychological assessment. In Gutkin T. B. Reynolds C. R. (Eds.), Mastering modern psychological testing (pp. 573–613). Springer.

[bibr35-00131644241237502] Samejima F. (1969). Estimation of latent ability using a response pattern of graded scores. Psychometrika Monograph Supplement, 34, 1–97.

[bibr36-00131644241237502] Schermelleh-Engel K. Moosbrugger H. Müller H. (2003). Evaluating the fit of structural equation models: Tests of significance and descriptive goodness-of-fit measures. Methods of Psychological Research Online, 8(2), 23–74.

[bibr37-00131644241237502] Scherpenzeel A. C. (2018). “True” longitudinal and probability-based internet panels: Evidence from the Netherlands. In Das M. Ester P. Kaczmirek L. (Eds.), Social and behavioral research and the internet (pp. 77–104). Routledge.

[bibr38-00131644241237502] Shmueli G. (2010). To explain or to predict? Statistical Science, 25(3), 289–310.

[bibr39-00131644241237502] Steyer R. Eid M. (2013). Messen und testen [Measuring and Testing]. Springer-Verlag.

[bibr40-00131644241237502] Strobl C. Kopf J. Zeileis A. (2015). Rasch trees: A new method for detecting differential item functioning in the Rasch model. Psychometrika, 80(2), 289–316. 24352514 10.1007/s11336-013-9388-3

[bibr41-00131644241237502] ten Holt J. C. van Duijn M. A. Boomsma A. (2010). Scale construction and evaluation in practice: A review of factor analysis versus item response theory applications. Psychological Test and Assessment Modeling, 52, 272–297.

[bibr42-00131644241237502] Van De Schoot R. Schmidt P. De Beuckelaer A. Lek K. Zondervan-Zwijnenburg M. (2015). Measurement invariance. Frontiers in Psychology, 6, Article 1064. 10.3389/fpsyg.2015.01064 PMC4516821 26283995

[bibr43-00131644241237502] Wager S. Athey S. (2018). Estimation and inference of heterogeneous treatment effects using random forests. Journal of the American Statistical Association, 113(523), 1228–1242.

[bibr44-00131644241237502] Wang T. Merkle E. C. Zeileis A. (2014). Score-based tests of measurement invariance: Use in practice. Frontiers in Psychology, 5, Article 438. 24936190 10.3389/fpsyg.2014.00438 PMC4038958

[bibr45-00131644241237502] Xi X. (2010). How do we go about investigating test fairness? Language Testing, 27(2), 147–170.

[bibr46-00131644241237502] Zeileis A. Hornik K. (2007). Generalized m-fluctuation tests for parameter instability. Statistica Neerlandica, 61(4), 488–508.

[bibr47-00131644241237502] Zeileis A. Hothorn T. Hornik K. (2008). Model-based recursive partitioning. Journal of Computational and Graphical Statistics, 17(2), 492–514.

